# Influence of the COVID-19 Lockdown and Restart on the Injury Incidence and Injury Burden in Men’s Professional Football Leagues in 2020: The UEFA Elite Club Injury Study

**DOI:** 10.1186/s40798-022-00457-4

**Published:** 2022-05-13

**Authors:** Markus Waldén, Jan Ekstrand, Martin Hägglund, Alan McCall, Michael Davison, Anna Hallén, Håkan Bengtsson

**Affiliations:** 1 Football Research Group, Linköping, Sweden; 2 Unit of Public Health, Department of Health, Medicine and Caring Sciences, Linköping University, Linköping, Sweden; 3 Unit of Physiotherapy, Department of Health, Medicine and Caring Sciences, Linköping University, Linköping, Sweden; 4 School of Applied Sport and Exercise Sciences, Edinburgh Napier University, Edinburgh, UK; 5 Arsenal Performance and Research Team, Arsenal Football Club, London, UK; 6 Isokinetic Medical Group, FIFA Medical Centre of Excellence, London, UK

**Keywords:** COVID-19, Epidemiology, Football, Injury burden, Injury incidence, Pandemic, Professional, Soccer

## Abstract

**Background:**

Studies on football and the coronavirus disease 2019 (COVID-19) have mainly focused on the lockdown consequences for player fitness, the resumption of football training, and how to safely restart the league play, but injury data are scarce.

**Objective:**

To describe the injury incidence and injury burden in men’s professional football teams during the pandemic year of 2020**.**

**Methods:**

Nineteen teams in 12 countries prospectively registered data on player-exposure and time-loss injuries throughout 2020. All major football leagues were paused as a direct response to the pandemic in March 2020 and were thereafter completely cancelled or restarted after a lockdown interval of at least two months. Historical data from 43 teams in the same cohort during the five preceding years (2015–2019) were used as reference. Between-season and within-season comparisons were made for injury incidence (number of injuries per 1000 h) and injury burden (number of absence days per 1000 h) with 95% confidence intervals and interquartile ranges.

**Results:**

There was no increased match injury incidence or injury burden following the restart in 2020 compared with other time periods of 2020 and the corresponding periods 2015–2019. There was an increased training injury incidence and injury burden immediately during the lockdown in 2020, and they remained elevated also following the restart, being higher in 2020 compared with 2015–2019, respectively. The injury characteristics during the first months of the new 2020/21 season (August/September–December) were similar between the five teams that cancelled their 2019/20 season in March 2020 and the 14 teams that restarted their season in May/June 2020.

**Conclusions:**

There was no increased match injury incidence or injury burden following the COVID-19 lockdown and restart of the football season in 2020, but training injury incidence and injury burden were elevated and higher than in 2015–2019.

## Key Points

- There were no increased match injury incidence and match injury burden following the restart of the men’s professional football leagues in 2020 compared with other time periods of 2020 and the corresponding periods in the 2015–2019 seasons.
- There were an increased training injury incidence and training injury burden immediately during the lockdown in 2020 and they remained high following the restart and the rest of the 2019/20 season.
- The injury patterns during the first months of the new 2020/21 season were similar between teams that cancelled their 2019/20 season following the lockdown in March 2020 and teams that restarted their season in May/June 2020.

## Background

In December 2019, there was a series of unexplained pneumonia cases in the city of Wuhan, China, and in February 2020 this new zoonotic virus disease was given the formal names coronavirus disease 2019 (COVID-19) and severe acute respiratory syndrome coronavirus 2 (SARS-CoV-2) [1, 2]. The COVID-19 pandemic was declared by the World Health Organization (WHO) on March 11, 2020, at which point the vast majority of the world’s major sports leagues and tournaments were interrupted, postponed or cancelled [3], including the European professional men’s leagues 2019/20, the Union of European Football Associations (UEFA) men’s Champions League 2019/20, the men’s UEFA Europe League 2019/20, as well as the UEFA men’s European Championship 2020.

Most of the current literature on professional football and COVID-19 has focused on the lockdown consequences for player fitness, how to manage the resumption of football training, and how to safely restart the league play [4–19]. To our knowledge, there are so far only one prospective study and five retrospective studies, with conflicting findings, that have sought to compare injury rates before and after the COVID-19 lockdown [20–25].

The UEFA Elite Club Injury Study (ECIS) was established in 2001 and is currently the largest scientific database of injuries in men’s professional football globally [26]. The injury data collection in the ECIS has remained consistent during the COVID-19 pandemic and is in a highly reputable position to provide a picture of the injury landscape before, during and after the COVID-19 lockdown with data collected directly through clubs. The objective of this study was, therefore, to describe the injury incidence and injury burden in men’s professional football teams during the year 2020 of the COVID-19 pandemic, including a comparison with the five preceding years 2015–2019 as reference.

## Materials and Methods

This study was performed in accordance with the standards of ethics outlined in the Declaration of Helsinki. Written informed consent was collected from all participating players before inclusion. The ECIS protocol has been approved by the UEFA Football Development Division and the UEFA Medical Committee.

### Study Procedure

The basic methodology of the ECIS has been essentially consistent since 2001 and will, in the interest of brevity, only be briefly summarized below [27]. The overall study design and the accompanying definitions adhere to international consensus statements on how to conduct studies and report injury data in epidemiological research in sports [28, 29].

At the start of the season, all participating teams assign one contact person in the club medical team to be responsible for all data collection. Club medical teams are provided with a study manual with detailed description of data collection procedures, operational definitions and the standard forms used in the study.

Data on football exposures and injury occurrences are reported monthly. Training and match exposures are registered on a daily basis in minutes of participation for each individual player. If players participated in training sessions or matches outside the first team, such as with the reserve teams or national teams, these exposures were also included. All player absences from training sessions and matches due to injury, illness, national team duty, or other reasons were also recorded.

Any injury occurrences are documented on a daily basis and reported monthly together with the exposure report. The one-page injury form contains data such as the date of the incident (or the player being taken out of training or matches), injury location and injury type, injury mechanism and other circumstances of injury. Injury was defined according to time-loss as any physical complaint sustained by a player that resulted from a football match or football training and led to the player being unable to take full part in future football training or match play. A player was considered injured until the club medical team considered the player ready for full participation in all team training activities and being available for match selection. Injury severity was defined as the number of days between the injury date and the date when the player was considered medically ready for full participation in all team activities. All data were reviewed by members of the study group to ensure that it complied with the study protocol. If any missing or unclear data were identified during this review process, immediate feedback was sent to the contact person to complete or correct the data.

### Study Population and Study Period

The primary study cohort consists of male professional players from 19 premier division teams in 12 countries that participated in the ECIS during both the 2019/20 and the 2020/21 seasons, thereby registering data throughout all of 2020. If a player left the team due to, for example, loan or transfer, data from that player were included during the time spent with the team. As a reference comparison to the pandemic year 2020, historical data from the five preceding years (2015–2019) of the ECIS were included from 43 teams in 18 European countries. These data were prospectively collected with the same methodology.

### Overview of Disruptions to Normal Football Season

In most European countries, the football season starts in July with a pre-season focused on training and friendly matches. The competitive season then typically starts in August and ends in May (starting and closing dates of campaigns vary slightly between countries). The 2019/20 and 2020/21 seasons were atypical due to the COVID-19 pandemic. While the 2019/20 season had started as normal, all major European football leagues were temporarily paused in March 2020 as a direct response to the developing COVID-19 pandemic. Switzerland was the first European country out on March 2, whereas the rest of the major European leagues followed between March 9 and 19 [30]. As a response to this, all 19 ECIS teams, except one, also stopped their team training with zero training hours reported for the rest of March with dates varying between teams from March 9 to March 17. Most football leagues then decided to continue their 2019/20 season, which applied for 14 of 19 teams participating in the ECIS. Training was initially carried out under atypical conditions often including home quarantine first and a severely restricted ability to train with a full squad on site later [9, 15, 19].

The German Bundesliga was the first league to restart on May 16 following a match-free interval of over two months and was followed by other European leagues such as the Spanish LaLiga on June 11, the English Premier League on June 17 and the Italian Serie A on June 20—all behind closed doors and with strict training, match and SARS-CoV-2 testing protocols [30]. Resumption of training for the teams that continued their season varied from April 1 to May 23 depending on the national lockdown lengths and different governmental restrictions. In some countries, such as France, Belgium and the Netherlands, the 2019/20 season was cancelled following the break in March, which applied for 5 of 19 teams in the ECIS. Any training exposure and training-related injuries were still recorded up to the off-season and start of the new pre-season 2020/21 for these teams. The final of the Champions League, which typically marks the end of the competitive season for all leagues, was played on August 23, three months later than originally planned, thus causing a delayed start of the 2020/21 season for the seven ECIS teams being involved in the knockout stage starting with the round of 16 leg matches in August 7 to August 8, 2020. Consequently, the starting dates for the new season for these teams varied from August 23 to September 11, whereas it started between June 22 and August 24 for the other twelve teams.

### Statistical Analysis

In general, data are reported for all teams, regardless if their season was cancelled or restarted, for the full year 2020 compared with 2015–2019. The injury incidence and injury burden for teams that had their seasons cancelled and teams that continued their seasons following the COVID-19-induced break are presented for three specific time periods: (i) the first three months of 2020, (ii) the restart of the 2019/20 season and (iii) the start of the new 2020/21 season. Data from similar time periods are also presented for the five preceding years (2015–2019). The start of the new 2020/21 season included the months September to December for teams that continued their 2019/20 season, and August to December for teams that had their 2019/20 season cancelled, and also for the 2015–2019 seasons.

Two different outcomes were calculated and reported for training and match play separately: injury incidence and injury burden. Injury incidence is reported as the number of injuries per 1000 h [(Σ injuries/Σ exposure hours) × 1000] with a corresponding 95% confidence interval (CI). The injury incidence during 2020 is compared with the injury incidence during 2015–2019 with a rate ratio (RR) and corresponding 95% CI.

Injury burden is reported as the number of injury absence days per 1000 h [(Σ absence days/Σ exposure hours) × 1000] with the corresponding interquartile range (IQR). In addition to aggregated data, the IQR is presented for both injury incidence and injury burden in tables and figure to illustrate the spread of the data. For 2020, IQR is presented as the first and fourth quartiles of all included teams. For 2015–2019, the IQR is presented as the first and fourth quartiles of the five included years.

## Results

### Injury Incidences in 2020 Versus 2015–2019

There were 690 injuries (342 training and 348 match play) during 114 533 exposure hours (97 987 training and 16 546 match play) during 2020. The total injury incidence was 6.0 (95% CI 5.6–6.5) injuries per 1000 h, with 3.5 (95% CI 3.1–3.9) injuries per 1000 training h and 21.0 (95% CI 18.9–23.4) injuries per 1000 match h. Between 2015 and 2019, a total of 5620 injuries (2425 training and 3195 match play) were reported during 1 040 904 exposure hours (887 491 training and 153 413 match play) representing a total injury incidence of 5.4 (95% CI 5.3–5.5) injuries per 1000 h, with 2.7 (95% CI 2.6–2.8) injuries per 1000 training h and 20.8 (95% CI 20.1–21.6) injuries per 1000 match h. As shown in Table 1, the injury incidence in training was thus 28% higher in 2020 compared to the average over the five preceding years (RR 1.28; 95% CI 1.14–1.43), while no difference was found for match injury incidence (RR 1.01; 95% CI 0.90–1.13).

**Table 1:** Injury incidence and injury burden for teams that had their 2019/20 season postponed and cancelled following the COVID-19 lockdown with corresponding data from 2015–2019

Period of season	2019/20 season postponed (14 teams)	2019/20 season cancelled (5 teams)	2015–2019 (mean (SD) 34 ± 3 teams)
*January to March 2020*			
Training injury incidence (IQR)	3.0 (1.9–3.3)	3.4 (2.5–4.6)	2.8 (2.7–2.9)
Match injury incidence (IQR)	22.5 (16.8–24.2)	24.0 (4.6–31.7)	22.0 (20.5–23.1)
Training injury burden (IQR)	79 (30–155)	33 (23–49)	51 (48–60)
Match injury burden (IQR)	582 (324–922)	457 (181–545)	426 (422–427)
Restart of the 2019/20 season*			
Training injury incidence (IQR)	4.1 (3.2–6.1)	N/A	3.0 (2.4–3.3)
Match injury incidence (IQR)	20.0 (11.0–22.2)	N/A	20.4 (17.9–22.6)
Training injury burden (IQR)	124 (69–191)	N/A	42 (38–43)
Match injury burden (IQR)	355 (182–508)	N/A	452 (394–515)
*Start of the 2020/21 season*^†^			
Training injury incidence (IQR)	3.3 (2.5–4.2)	3.7 (3.4–4.2)	2.7 (2.5–3.0)
Match injury incidence (IQR)	21.0 (14.8–34.0)	19.8 (15.7–19.4)	21.1 (20.6–21.3)
Training injury burden (IQR)	56 (38–74)	62 (27–79)	57 (57–67)
Match injury burden (IQR)	449 (158–692)	532 (365–632)	534 (534–555)
*Full calendar year*			
Training injury incidence (IQR)	3.4 (2.7–4.1)	3.6 (3.1–3.8)	2.7 (2.7–2.9)
Match injury incidence (IQR)	21.2 (16.6–24.6)	20.6 (11.4–21.9)	20.8 (20.5–21.4)
Training injury burden (IQR)	83 (61–103)	59 (54–65)	52 (45–59)
Match injury burden (IQR)	469 (331–665)	485 (402–461)	484 (476–509)

Injury incidence is presented as the number of injuries per 1000 h and injury burden as the number of injury absence days per 1000 h. Injury incidence is presented with one decimal and injury burden (days) without

IQR, interquartile range; SD, standard deviation

*April to July for teams that had their 2019/20 season postponed and April–May for 2015–2019

^†^September to December for teams that had their 2019/20 season postponed and August to December for both teams that had their 2019/20 season cancelled and for teams 2015–2019

There were 15 337 absence days due to injury (7518 training days and 7819 match days) during 2020. The total injury burden was 134 absence days per 1000 h (IQR 99–170), with 77 absence days per 1000 training hours (IQR 57–101) and 473 absence days per 1000 match hours (IQR 331–665). A total of 120 318 days of absence due to injury were reported between 2015 and 2019 representing a total injury burden of 116 absence days per 1000 h (IQR 103–131), with 52 absence days per 1000 training hours (IQR 44–61) and 484 absence days per 1000 match hours (IQR 458–512).

### Injury Incidences in the Different Time Periods of 2020

**Fig. 1:**
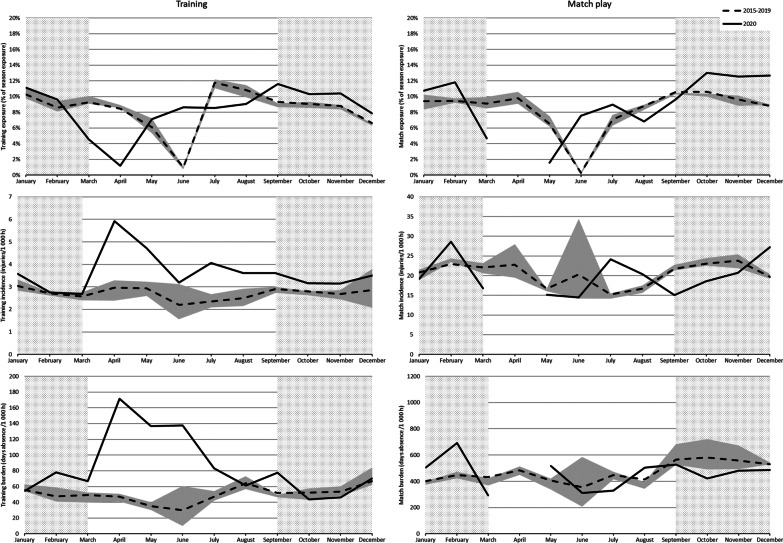
Monthly exposure, injury incidence and injury burden in European football clubs during 2020 compared with historical data 2015–2019. Injury incidence is presented as the number of injuries per 1000 h, and injury burden is presented as the number of injury absence days per 1000 h. The interquartile ranges for the period 2015–2019 are represented by the shaded areas

Monthly exposure, injury incidence and injury burden are presented in Fig. 1. The figure indicates a surge with an increased training injury incidence and training injury burden in April 2020 during the lockdown period compared with April 2015–2019. These high levels remained elevated during the rest of the 2019/20 season including the restart before returning to normal when the 2020/21 competitive season started in August/September 2020. In contrast, no apparent differences in match injury incidence and match injury burden were seen between 2020 and previous seasons.

Injury incidence and injury burden during different periods of 2020 for teams that had their 2019/20 season postponed and teams that had their 2019/20 season cancelled are presented in Table 1. Teams that continued their 2019/20 season following the COVID-19 lockdown reported a higher training injury incidence and training injury burden during the lockdown and restart period compared with the other time periods of 2020 as well as with previous seasons. Particularly, the training injury burden was increased during this period and almost threefold to what has been reported in the corresponding season-end period of previous seasons.

The injury pattern during the different time periods of 2020 and for 2015–2019 is presented in Table 2. The injury pattern during the first months of the 2020/21 season was similar between teams that cancelled their season and teams that restarted their season with just over 50% muscle injuries and approximately three-quarters of all injuries reported as non-contact injuries.

**Table 2:** Injury pattern for teams that had their 2019/20 season postponed and cancelled following the COVID-19 lockdown with corresponding data from 2015–2019

Period of season	2019/20 season postponed (522 injuries)	2019/20 season cancelled (168 injuries)	2015–2019 (5620 injuries)
*January to March 2020*			
Injury type			
Muscle injuries	61 (48%)	26 (50%)	757 (46%)
Ligament injuries	20 (16%)	11 (21%)	215 (13%)
Other injury types	47 (37%)	15 (29%)	667 (41%)
Injury mechanism			
Contact	29 (23%)	14 (27%)	437 (27%)
Non-contact	99 (77%)	38 (73%)	1202 (73%)
Injury severity			
Slight/Minimal (0–3 days)	18 (14%)	6 (12%)	246 (15%)
Mild (4–7 days)	30 (23%)	15 (29%)	393 (24%)
Moderate (8–28 days)	53 (41%)	26 (50%)	698 (43%)
Severe (> 28 days)	27 (21%)	5 (10%)	302 (18%)
Restart of the 2019/20 season*			
Injury type			
Muscle injuries	75 (52%)	N/A	406 (46%)
Ligament injuries	21 (15%)	N/A	138 (15%)
Other injury types	47 (33%)	N/A	347 (39%)
Injury mechanism			
Contact	31 (22%)	N/A	232 (26%)
Non-contact	112 (78%)	N/A	659 (74%)
Injury severity			
Slight/Minimal (0–3 days)	22 (15%)	N/A	133 (15%)
Mild (4–7 days)	33 (23%)	N/A	238 (27%)
Moderate (8–28 days)	65 (45%)	N/A	394 (44%)
Severe (> 28 days)	23 (16%)	N/A	126 (14%)
*Start of the 2020/21 season*^†^			
Injury type			
Muscle injuries	125 (57%)	55 (55%)	1 214 (46%)
Ligament injuries	23 (11%)	11 (11%)	453 (17%)
Other injury types	71 (32%)	34 (34%)	985 (37%)
Injury mechanism			
Contact	47 (21%)	28 (28%)	750 (28%)
Non-contact	172 (79%)	72 (72%)	1 902 (72%)
Injury severity			
Slight/Minimal (0–3 days)	25 (11%)	10 (10%)	352 (13%)
Mild (4–7 days)	47 (21%)	30 (30%)	534 (20%)
Moderate (8–28 days)	110 (50%)	42 (42%)	1 184 (45%)
Severe (> 28 days)	37 (17%)	18 (18%)	582 (22%)
*Full calendar year*			
Injury type			
Muscle injuries	278 (53%)	89 (53%)	2 564 (46%)
Ligament injuries	68 (13%)	24 (14%)	885 (16%)
Other injury types	176 (34%)	55 (33%)	2 171 (39%)
Injury mechanism			
Contact	113 (22%)	46 (27%)	1 541 (27%)
Non-contact	409 (78%)	122 (73%)	4 079 (73%)
Injury severity			
Slight/Minimal (0–3 days)	69 (13%)	21 (13%)	831 (15%)
Mild (4–7 days)	115 (22%)	47 (28%)	1 253 (22%)
Moderate (8–28 days)	241 (46%)	74 (44%)	2 438 (43%)
Severe (> 28 days)	97 (19%)	26 (15%)	1 098 (20%)

*April to July for teams that had their 2019/20 season postponed and April–May for 2015–2019

^†^September to December for teams that had their 2019/20 season postponed and August to December for teams that had their 2019/20 season cancelled and for 2015–2019

## Discussion

Our study is the first to take training exposure and training injuries into consideration when studying the influence of the COVID-19 lockdown and restart of the football season on the injury incidence and injury burden in men’s professional football. The principal finding was that there was no increased match injury incidence or match injury burden following the COVID-19 lockdown and restart in 2020. A spike in the training injury incidence and training injury burden was, however, identified in April 2020 compared with the same period during the preceding five seasons 2015–2019. Finally, the injury pattern during the first months of the new 2020/21 season was similar between teams that cancelled their 2019/20 season following the lockdown in March 2020 and teams that restarted their season in May/June 2020.

### Injuries During the Pandemic 2020

In order to study the potential influence of the COVID-19 lockdown and restart of the football season, a pragmatic approach was used with comparisons between three distinct time periods of 2020 and the full calendar year of 2020 with the aggregate values of the five preceding years 2015–2019 (considered as normal football calendars). Similar to the findings in a recent small retrospective study on the Norwegian professional first league (Tippeligan) 2020 compared with 2019 [20], we did not find an increased match injury incidence and match injury burden in 2020 compared with 2015–2019. Whereas the Norwegian study only investigated match injuries in eight teams retrospectively, our study used prospectively collected data and had a considerably larger sample (19 teams). Additionally, by using the average measures of the five preceding seasons as reference instead of just the preceding regular season, we were able to consider the possible impact of any normal inter-season variations and this approach is also statistically more robust than using data from a single season before the pandemic which could be an “outlier” (up or down) season. Similarly, another study on eleven of the 20 teams in the Spanish LaLiga, using prospectively recorded data, identified no difference in the overall injury incidence between the pre-lockdown competitive period from the league start in August 16, 2019, and the post-lockdown period from June 11, 2020 [23]. Our findings were also fairly similar to another recent retrospective study on the German Bundesliga which used publicly available data for comparing the injury statistics from the 9-match restart period 2019/2020 with the preceding calendar year [21]. That study found a lower injury incidence in the final nine match days following the restart 2020 than for the same match period the preceding season 2019, and the authors speculate that this might be due to cure of long-standing overuse-related injuries or other minor injuries perhaps predisposing to other subsequent major injuries as well as a possibility for increased and more individualized preventive training during the lockdown. Another approach was taken in a retrospective study on the French Ligue 1 and 2, where the 2020/21 season, characterized by a longer pre-season and shorter league duration following the cancellation of the 2019/20 season, was compared with a regular season 2018/19 [24]. Interestingly, that study found a lower match injury incidence in 2020/21 than in 2018/19, especially for Ligue 1 teams which also had a lower incidence of muscle strains.

In contrast, two other retrospective studies on the German Bundesliga 2019/20 and the English Premier League 2018/19–2020/21, using only publicly available data, reported more injuries than with their control periods. The Bundesliga study reported a significantly higher injury rate per match after the restart (May 16 to June 28, 2020) compared with the pre-lockdown period starting from the August 16, 2019 [25], and the Premier League study reported more absolute numbers of muscular and ligamentous injuries in 2020/21 compared with the two preceding seasons 2018/19 and 2019/20, but with no exposure factor accounted for [22]. None of the previous studies have investigated training exposure and training injuries separately which we did in the current study. Interestingly, therefore, we identified an increased training injury incidence and training injury burden immediately during the lockdown in April 2020, measures which remained elevated for the rest of the 2019/20 season, and they were also higher compared with the historical data from 2015 to 2019. The underlying reasons are unclear, but it could be speculated that the lockdown period allowed medical teams to rest and treat players with long-standing overuse-related injuries and that the restart period was characterized by more match-like training protocols to increase or maintain player fitness in the absence of friendly matches before competitive matches [21].

### Resumption of Training and Return to Play Protocols

The scientific, football, media and social media community all anticipated a higher match injury incidence and match injury burden following the lockdown and restart of the football season, particularly in terms of muscle injuries and other non-contact injuries. The players, coaches and medical staff were faced with a new normality, and the return to the club training environments after the initial lockdown period, with limited access to coaches and medical staff [9], was a complete unknown and resembled the situation of the National Football League (NFL) lockdown in May to July, 2011 [31]. This was shaped by the international and national training resumption rules and recommendations associated with the prevention of transmission of the virus [9, 13, 15, 19].

The beginning of the new competitive 2020/21 season, with its shorter pre-season period than usual, was anticipated with having potential spikes in injuries [32], but we did not see any notable variations during training or match play at the restart of the 2020/21 season including muscle injuries and non-contact injuries. Despite the severe disruptions caused by the lockdown and the challenging circumstances faced by team medical and performance practitioners, they had, however, plenty of time to plan for the start of the 2020/21 season. Internal communication quality has recently been shown to be a factor to consider from an injury perspective [33], and the pandemic has forced normal communication channels in place to change and the level of internal communication, in relation to injury prevention and physical preparation topics, was likely to have been prioritized during this period.

### Methodological Considerations

This study is strengthened by the prospective design of the data collection and by the fact that the study has been running for several years, using the same methodology, allowing for comparisons with historic data registered before the COVID-19 pandemic developed. Our study also took training exposure and training injuries into considerations which previous retrospective studies on the pandemic and injuries in professional football have not [20–25]. Additionally, the study design and definitions adhere to consensus about how to conduct epidemiological studies in football [27, 29].

Some limitations should, however, also be acknowledged. First, although a relatively large sample was included, teams represented several different football associations and the number of teams from each association were few, occasionally just one team per association included. Consequently, although there might be lockdown length-related differences in physical performance of players [19], and in turn perhaps also for injuries, this was not possible to study in detail to respect team confidentiality. Second, government rules and return to play recommendations in response to the COVID-19 pandemic differed between associations [13, 19], and we did not have access to all the football resumption protocols. Third, no detailed information about the training protocols of the teams, including the type of training or the individual training workload, was registered similar to previous studies [20–25]. Potential associations between the increased injury incidence in training that were observed following the lockdown and potential changes in training protocols due to the COVID-19 pandemic therefore remain speculative and need further study. Fourth, thresholds for removing players from training due to complaints might have been different during the lockdown which is important to take into consideration because we used a time-loss injury definition [27–29]. Fifth, due to the team inclusion criteria and outgoing player contracts, loans, transfers, etc., players come and go to the ECIS and can thus participate for parts of a season, a full season, several seasons on an irregular basis and several consecutive seasons. No adjustments for this were made in the analyses which used team-based data or aggregated data exclusively. Finally, although absence for other reasons than injury, such as illness, is also documented on the exposure report form, there was no accompanying illness card so data on the number of players with COVID-19, symptomatic and asymptomatic, are incomplete and could not be included in the analyses.

## Conclusions

In this prospective cohort study on men’s professional football teams in Europe, there was no increased match injury incidence or match injury burden following the COVID-19 lockdown in March 2020 and the restart in May/June 2020. However, a spike in the training injury incidence and training injury burden was identified in April 2020 during the lockdown compared with the same period during the preceding five seasons 2015–2019. Finally, the injury patterns during the first months of the new 2020/21 season were similar between teams that cancelled their 2019/20 season following the lockdown in March 2020 and teams that restarted their season in May/June 2020.

## Data Availability

The datasets generated and/or analyzed during the current study are not publicly available due to being held securely in coded form, but are available in coded form from the corresponding author on reasonable request.

## Abbreviations

COVID-19: Coronavirus disease 2019
CI: Confidence interval
ECIS: Elite Club Injury Study
IQR: Interquartile range
RR: Rate ratio
SARS-CoV-2: Severe acute respiratory syndrome coronavirus 2
SD: Standard deviation
UEFA: Union of European Football Associations
WHO: World Health Organization
